# Elevated risk of hepatitis E virus infection among sheep smallholders in Xinjiang, China

**DOI:** 10.1016/j.onehlt.2026.101361

**Published:** 2026-02-15

**Authors:** Wan-Ting Xu, Yu-Song Ding, Yang-Gui Chen, Ya-Hui Feng, Jun Li, Ji-Guo Jin, Xiang-Nan Wei, Fan Wu, Xing-Yu Wang, Xing-Tao Dang, Guo-Wu Zhang, Xue-Ying Xiang, Fu-Ye Li, Wen-Bao Zhang, Jian-Yong Wu

**Affiliations:** a Key Laboratory of Special Environment and Health Research in Xinjiang, School of Public Health, Xinjiang Medical University, Urumqi 830017, China; b Key Laboratory of Environmental Exposure Omics, Institute of Medical Sciences, Xinjiang Medical University, Urumqi 830017, China; c Urumqi Center for Disease Control and Prevention, Urumqi 830026, China; d State Key Laboratory of Pathogenesis, Prevention and Treatment of High Incidence Diseases in Central Asia, Clinical Medicine Institute, The First Affiliated Hospital of Xinjiang Medical University, Urumqi 830052, China

**Keywords:** Hepatitis E virus, Seroprevalence, Occupational risk, Sheep worker, One health

## Abstract

Hepatitis E virus (HEV) is an emerging zoonotic pathogen that infects both humans and domestic animals. Although HEV has been detected in sheep, the risks of transmission along the food chain via the mutton industry chain remain poorly characterized. This study aimed to assess the occupational risk of HEV infection among sheep industry workers. A cross-sectional survey was conducted among sheep workers in various settings, including smallholder farms, slaughterhouses, and retail markets. Serum samples from these workers, as well as from individuals in the general population, were collected and tested for anti-HEV IgM and IgG antibodies using commercial ELISA kits. Risk factors for HEV seropositivity across different occupational environments were analyzed using logistic regression. The seropositivity rate among sheep industry workers was 53.0% (132/249), significantly higher than that in the general population (13.3%, 33/249). Multi-variable analysis revealed a markedly increased risk of HEV infection among smallholder sheep farmers (adjusted OR = 10.1, 95% CI: 6.3–16.3). In contrast, there was no significantly elevated risk among people working in retail markets or slaughterhouses. The results indicate that occupational exposure to sheep is associated with a heightened risk of HEV infection, particularly in smallholder farm settings. Strengthening HEV control measures for HEV in high-risk occupational environments is essential for long-term public health prevention and elimination efforts.

## 1 Introduction

Hepatitis E virus (HEV) is an emerging pathogen that causes acute viral hepatitis [1]. As an RNA virus, HEV can infect a wide range of hosts, including humans, pigs, wild boars, deer, and rabbits [2]. Although HEV typically causes self-limiting hepatitis in humans, it can lead to severe clinical outcomes, including chronic hepatitis in immunocompromised individuals and high mortality rates among pregnant women in developing countries [[3], [4], [5]]. Transmission to humans occurs primarily through fecal–oral, blood transfusion and zoonotic routes, with the latter including the consumption of raw or undercooked meat products, as well as direct exposure to infected animals [6,7]. HEV is responsible for an estimated 20 million infections annually, resulting in approximately 3.4 million symptomatic cases and over 70,000 deaths [8]. These figures highlight the substantial public health burden imposed by the virus.

HEV is classified in the subfamily *Orthohepevirinae* that comprises four genera: *Avihepevirus*, *Chirohepevirus*, *Paslahepevirus*, and *Rocahepevirus*. Among these, *Paslahepevirus* and *Rocahepevirus* (which includes rat HEV) are known to infect humans [9]. The species *Paslahepevirus balayani,* in the genus *Paslahepevirus,* is the main causative agent of hepatitis E in humans and comprises eight genotypes (HEV-1 to HEV-8) [10]. Genotypes HEV-1 and HEV-2 are obligate human pathogens and are primarily transmitted via contaminated water in endemic regions [11]. In contrast, HEV-3 and HEV-4 are zoonotic genotypes capable of infecting both humans and animals, including pigs and wild boars, with rabbits serving as important reservoirs for HEV-3 [[12], [13], [14]]. These zoonotic genotypes are significant public health concerns due to their ability to be transmitted through consumption of undercooked meat or direct contact with infected animals [15]. In addition, HEV-5 and HEV-6 have been identified in wild boars, while HEV-7 and HEV-8 have been found in camels. Occasional transmission of HEV-7 to humans has also been reported [[15], [16], [17]].

High anti-HEV seroprevalence has been reported among sheep and goats [[18], [19], [20]], and occupational exposure to these animals has been associated with an increased risk of HEV infection in humans, as demonstrated by the elevated seroprevalence among workers handling sheep [21]. However, there remains a notable scarcity of research systematically assessing infection risks among sheep industry workers across different occupational settings. The seroprevalence of HEV infection varies considerably among occupational groups. Veterinarians and workers exposed to swine in slaughterhouses and retail markets consistently exhibit higher antibody levels. This heightened seroprevalence is likely attributable to their more frequent exposure to the virus despite compliance with biosafety protocols [22,23]. This discrepancy underscores the need for more finely stratified occupational risk assessments. Workers on smallholder farms or in retail markets, for example, have limited access to protective equipment and often lack systematic training to mitigate zoonotic risk. Such information gaps may significantly heighten their vulnerability to HEV infection through direct animal contact or exposure to contaminated materials.

HEV is highly endemic in China, with a nationwide meta-analysis reporting an overall seroprevalence of 29.2% (95% CI: 26.0–32.4) among blood donors, indicating widespread viral circulation in the general population [24,25]. Occupational populations, such as pig farmers and slaughterhouse workers, exhibit markedly elevated seroprevalence, reinforcing the role of repeated animal exposure in HEV transmission [22]. Regional data further illustrate this point. Xinjiang, located in northwestern China, was the epicenter of a major HEV outbreak in the 1980s, during which a significant number of cases were reported, particularly among pregnant women [[26], [27], [28]]. Subsequent surveillance revealed high seroprevalence in livestock: 84.6% in slaughter pigs and 35.2% in sheep [27]. Even higher rates have been documented in wild boars, with one study reporting a prevalence of 70.0% [29]. HEV-4 had been detected in sheep in Xinjiang and other parts of northwestern China, suggesting that sheep may act as amplifying hosts in local transmission networks [18,20]. Molecular epidemiological evidence further indicates that sheep may be involved in local zoonotic transmission cycles [20]. These findings suggest significant variability in reservoir competence across animal species and collectively highlight the complex interplay between dietary behavior, occupational exposure, and ecological reservoir dynamics in shaping the epidemiology of HEV in high-endemicity settings.

Given the high prevalence of HEV infection in sheep, an important source of mutton and offal in northwest China, and considering the current lack of studies on sheep-associated occupational risks, there is an urgent need to evaluate the risk of HEV infection among individuals exposed to these animals. This study aims to investigate the seroprevalence of HEV infection among sheep industry workers across multiple settings, including smallholder farms, retail markets, and slaughterhouses. By comparing these findings with those from the general population, we seek to clarify occupation-related HEV risks and generate evidence to support targeted vaccination strategies and other protective measures for high-risk populations.

## 2 Materials and methods

### 2.1 Study design

The study was conducted from June 2023 to June 2025 in Xinjiang, Northwest China. Participants included sheep industry workers and individuals from the general population without exposure to livestock (including sheep, goats, cattle, camels, or pigs). A total of 249 sheep industry workers were recruited from various occupational settings, including smallholder farms, retail markets, and slaughterhouses. Most of these farmers raised sheep as a secondary source of income, with crop farming or small-scale trade being their primary occupation, and they reported no routine exposure to pigs or other HEV reservoir animals. The inclusion criteria were as follows: 1) having worked or lived in settings with sheep exposure to sheep (including working on a smallholder farm, in a slaughterhouses, or in a retail market) for more than six months, with exposure exceeding 10 h per week continuously for at least three months, and 2) being at least 18 years of age. Exclusion criteria included: 1) immunocompromised status, ongoing acute gastrointestinal tract infection, or pregnancy; 2) diagnosis of immunosuppressive or immunodeficiency diseases (including HIV infection) or current receipt of immunosuppressive therapy; 3) a history of HEV vaccination within the past 10 years, and 4) reported routine occupational or domestic exposure to pigs, cattle, camels, or other livestock known to be HEV reservoirs. A 3–5 mL blood sample was collected from each participant. Face-to-face interviews were conducted by trained personnel to gather demographic information, history of exposure to sheep, and other relevant details.

The study protocol, including blood sampling and face-to-face interviews, was reviewed and approved by the Ethics Committee of Xinjiang Medical University (approval no. XJYKDXR20230510012). Written informed consent was obtained from all of the participants prior to the collection of blood samples and administration of interviews.

### 2.2 Laboratory analysis

Commercial HEV enzyme-linked immunosorbent assay (ELISA) kits (Wantai BioPharm, Beijing, China) were employed to detect HEV-specific IgG and IgM antibodies. These kits utilize recombinant antigens from HEV-1, demonstrating broad cross-reactivity with antibodies against all four major human-pathogenic genotypes (HEV-1, HEV-2, HEV-3, and HEV-4), which are the most common in China [30]. The IgG assay exhibited a specificity of 99.90% and a sensitivity of 99.08%, while the IgM assay demonstrated a specificity of 98.40% and a sensitivity of 97.10%. All samples were tested in a single measurement according to the manufacturer's instructions. The cut-off value for determining seropositivity was calculated as follows: Cut-off = mean negative control optical density (OD) + Constant, where the constant was 0.16 for IgG and 0.26 for IgM. A sample was considered positive if its OD was equal to or greater than this calculated cut-off value.

### 2.3 Statistical analysis

The survey data were managed using EpiData 3.1 (http://epidata.dk/). All of the data were double-entered independently, and logical consistency checks were conducted using Structured Query Language (SQL) to ensure accuracy. The survey data and laboratory results were merged into a master dataset using unique participant identification numbers. Data analysis was performed using SPSS version 26.0 (IBM, Chicago, USA), R (version 4.5.1), and RStudio. Continuous variables following a normal distribution were presented as mean ± standard deviation (SD). Categorical variables were described as frequencies (percentages). HEV serological results were initially analyzed as dichotomous variables using two-sided chi-square tests, as appropriate. Propensity score matching was conducted in R with age and gender as covariates to perform 1:1 non-repetitive matching between occupational sheep industry workers and the general population (caliper value set to 0.3) to achieve balanced baseline characteristics between the two groups. Univariate logistic regression was used to identify factors associated with prior HEV infection, and multivariate logistic regression was used to assess occupational risk after adjusting for potential confounders such as age and gender. The analysis incorporated data from the general population, in which age served as a proxy for HEV infection, thereby allowing estimation of additional infection risk associated with sheep exposure to sheep.

## 3 Results

The study enrolled 249 sheep industry workers and 249 matched controls from the general population (Table 1). The mean age was 48.5 years (SD = 11.6) among the sheep industry workers and 50.6 years (SD = 13.2) among the controls, with no statistically significant difference between groups (Z = −1.608, *p* = 0.108). Age distribution across categorical groups (18–34, 35–44, 45–54, and ≥ 55 years) also did not differ significantly (χ^2^ = 2.560, *p* = 0.465). There was a higher proportion of males among the sheep industry workers (66.3%) compared to the general population (61.0%), although this difference was not statistically significant (χ^2^ = 1.467, *p* = 0.226). Among the sheep industry workers, the duration of exposure to sheep was distributed as follows: 0–4 years (30.1%), 5–14 years (37.8%), and ≥ 15 years (32.1%). The majority of workers were employed in smallholder farms (86.0%), followed by slaughterhouses (10.0%) and retail markets (4.0%).

**Table 1:** Comparative characteristics of sheep industry workers and the general populations.

Variable	Sheep industry worker (*n* = 249)	General population (*n* = 249)	*χ*^2^/*Z*	*p value*
Age (y, mean, SD)	48.5 (11.6)	50.6 (13.2)	−1.608	0.108
Age (y)			2.560	0.465
18-	32 (12.9)	29 (11.6)		
35-	59 (23.7)	46 (18.5)		
45-	66 (26.5)	71 (28.5)		
55-	92 (36.9)	103 (41.4)		
Gender			1.467	0.226
Male	165 (66.3)	152 (61.0)		
Female	84 (33.7)	97 (39.0)		
Duration of exposure to sheep (y)				
0-	75 (30.1)			
5-	94 (37.8)			
15-	80 (32.1)			
Workplace				
Retail market	10 (4.0)			
Smallholder farm	214 (86.0)^⁎^			
Slaughterhouse	25 (10.0)			

Note: ^⁎^ Totals may not equal 100% due to rounding, the largest component has been adjusted to reconcile the total.

The overall seroprevalence of anti-HEV IgG and total antibodies (HEV) was significantly higher among sheep industry workers (51.8% and 53.0%, respectively) compared to the general population (12.9% and 13.3%, respectively) (both *p* < 0.001). In contrast, no significant difference was observed in IgM seropositivity, with five (2.0%) sheep industry industry workers and one (0.4%) control individual testing positive. In addition, two sheep industry workers were seropositive for both IgG and IgM antibodies. Significantly higher seroprevalence was found in both male and female sheep industry workers compared to controls (all *p* < 0.001 for IgG and HEV). Notably, female workers had the highest observed IgG seroprevalence at 69.0%, compared to 14.4% in control females. No significant gender-based differences in IgM seropositivity were detected within or between the two populations. Analysis by workplace revealed that workers in smallholder farms had the highest seroprevalence (IgG: 56.5%, total antibodies: 57.9%), which was significantly greater than the general population (*p* < 0.001). Sheep industry workers in retail markets and slaughterhouses showed elevated but non-significant trends towards higher seroprevalence compared to controls.For several subgroup comparisons (e.g., IgM in specific age/gender groups), *p* values were not applicable or could not be reliably calculated due to zero positive cases in one or both groups. Despite sporadic IgM positivity, None of these IgM-positive participants reported acute hepatitis symptoms at enrollment (Table 2).

**Table 2:** the overall Seroprevalence of HEV infection in participates in this study.

Antibody Type	Group	IgG		*p* value	IgM		*p* value	HEV		*p* value
		Seroprevalence % (*n* / *N*)	(95% CI)		Seroprevalence % (*n* / *N*)	(95% CI)		Seroprevalence % (*n* / *N*)	(95% CI)	
Overall	Sheep industry workers	51.8 (129/249)	(45.6, 58.1)	<0.001	2.0 (5/249)	(0.7, 4.6)	0.100	53.0 (132/249)	(46.8, 59.3)	<0.001
	General populations	12.9 (32/249)	(8.7, 17.0)		0.4 (1/249)	(0.0, 2.2)		13.3 (33/249)	(9.0, 17.5)	
Age (y)										
18-	Sheep industry workers	18.8 (6/32)	(4.5, 33.0)	0.141	0 (0/32)	(0.0, 0.0)		18.8 (6/32)	(4.5, 33.0)	0.106
	General populations	3.4 (1/29)	(0.1, 17.8)		0 (0/29)	(0.0, 0.0)		3.4 (1/29)	(0.1, 17.8)	
35-	Sheep industry workers	44.1 (26/59)	(31.0, 57.1)	<0.001	0 (0/59)	(0.0, 0.0)		45.8 (27/59)	(32.7, 58.9)	<0.001
	General populations	8.7 (4/46)	(2.5, 19.7)		0 (0/46)	(0.0, 0.0)		8.7 (4/46)	(2.5, 19.7)	
45-	Sheep industry workers	68.2 (45/66)	(56.6, 79.7)	<0.001	0 (0/66)	(0.0, 0.0)	1.000	68.2 (45/66)	(56.6, 79.7)	<0.001
	General populations	7.0 (5/71)	(0.9, 13.1)		1.4 (1/71)	(0.0, 7.8)		8.5 (6/71)	(1.8, 15.1)	
55-	Sheep industry workers	56.5 (52/92)	(46.2, 66.8)	< 0.001	4.3 (4/92)	(1.2, 10.8)	0.103	58.7 (54/92)	(48.4, 68.9)	< 0.001
	General populations	21.4 (22/103)	(13.3, 29.4)		0 (0/103)	(0.0, 0.0)		21.4 (22/103)	(13.3, 29.4)	
Gender										
Male	Sheep industry workers	43.0 (71/165)	(35.4, 50.7)	< 0.001	2.4 (4/165)	(0.7, 6.1)	0.153	44.8 (74/165)	(37.2, 52.5)	<0.001
	General populations	11.8 (18/152)	(6.6, 17.0)		0 (0/152)	(0.0, 0.0)		11.8 (18/152)	(6.6, 17.0)	
Female	Sheep industry workers	69.0 (58/84)	(59.0, 79.1)	< 0.001	1.2 (1/84)	(0.0, 6.5)	1.000	69.0 (58/84)	(59.0, 79.1)	< 0.001
	General populations	14.4 (14/97)	(7.3, 21.6)		1.0 (1/97)	(0.0, 5.6)		15.5 (15/97)	(8.1, 22.8)	
Workplace										
Retail market	Sheep industry workers	30.0 (3/10)	(6.7, 65.3)	0.279	0 (0/10)	(0.0, 0.0)	1.000	30.0 (3/10)	(6.7, 65.3)	0.148
	General populations	as above								
Smallholder farm	Sheep industry workers	56.5 (121/214)	(49.8, 63.2)	<0.001	2.3 (5/214)	(0.3, 4.4)	0.155	57.9 (124/214)	(51.3, 64.6)	<0.001
	General populations	as above								
Slaughterhouse	Sheep industry workers	20.0 (5/25)	(3.1, 36.9)	0.490	0 (0/25)	(0.0, 0.0)	1.000	20.0 (5/25)	(3.1, 36.9)	0.362
	General populations	as above								

Note: CI, confidential interval.

The risk factor analysis identified several variables that were significantly associated with HEV seropositivity among sheep industry workers. Increasing age (OR = 1.06, 95% CI: 1.03–1.08), female gender (OR = 2.7, 95% CI: 1.6–4.8), and longer exposure duration to sheep were independent risk factors. Stratification by exposure duration showed that workers with 5–14 years of exposure had the highest infection risk (OR = 10.1, 95% CI: 5.8–17.5). Furthermore, sheep workers on smallholder farms were associated with markedly elevated seroprevalence (OR = 9.0, 95% CI: 5.7–14.2), whereas there was no significant association among those working in retail markets or slaughterhouses (Table 3).

**Table 3:** Seroprevalance and risk factors for HEV infection in sheep industry workers.

Variable	*N*	Seropositive *n*	Seroprevalence % (95% CI)	OR (95% CI)	*p* value
**Age (y)**				1.06 (1.03, 1.08)	<0.001
18-	32	6	18.8 (4.5, 33.0)	Reference	
35-	59	27	45.8 (32.7, 58.9)	3.7 (1.3, 10.2)	
45-	66	45	68.2 (56.6, 79.7)	9.3 (3.3, 26.0)	
55-	92	54	58.7 (48.4, 68.9)	6.2 (2.3, 16.4)	
Gender					
Male	165	74	44.8 (37.2, 52.5)	Reference	
Female	84	58	69.0 (59.0, 79.1)	2.7 (1.6, 4.8)	
**Duration of exposure to sheep (y)**					<0.001
General populations	249	33	13.3 (9.0, 17.5)	Reference	
0-	75	34	45.3 (33.8, 56.9)	5.4 (3.0, 9.7)	
5-	94	57	60.6 (50.6, 70.7)	10.1 (5.8, 17.5)	
15-	80	41	51.3 (40.1, 62.4)	6.9 (3.9, 12.2)	
**Workplace**					<0.001
General populations	249	33	13.3 (9.0, 17.5)	Reference	
Retail market	10	3	30.0 (6.7, 65.3)	2.8 (0.7, 11.4)	
Smallholder farm	214	124	57.9 (51.3, 64.6)	9.0 (5.7, 14.2)	
Slaughterhouse	25	5	20.0 (3.1, 36.9)	1.6 (0.6, 4.7)	

Note: OR, odds ratio; CI, confidential interval.

After adjusting for potential confounders including age and gender, sheep industry workers exhibited an aOR of 9.5 (95% CI: 5.9–15.3) for HEV seropositivity compared to the general population, and the difference was highly significant (*Wald* χ^2^ = 86.004, *p* < 0.001; Table 4). Furthermore, the risk of HEV infection among individuals from smallholder farms varied significantly across different occupational settings (*Wald* χ^2^ = 90.058, *p* < 0.001; Table 4). Compared to the general population, retail workers had an aOR of 4.8 (95% CI: 1.1–21.1; *Wald* χ^2^ = 4.339, *p* = 0.037). The aOR was considerably higher among smallholder farmers (aOR = 10.1; 95% CI: 6.3–16.3), while slaughterhouse workers had an aOR of 4.3 (95% CI: 1.3–13.8). All of these associations were statistically significant.

**Table 4:** Occupational risk of HEV infection in sheep industry workers.

Variable	*N*	Seropositive *n*	Seroprevalence % (95% CI)	aOR (95% CI)	*Wald*	*p* value
Occupation					86.004	<0.001
General populations	249	33	13.3 (9.0, 17.5)	Reference		
Sheep industry workers	249	132	53.0 (46.8, 59.3)	9.5 (5.9, 15.3)		
**Workplace**					90.058	< 0.001
General populations	249	33	13.3 (9.0, 17.5)	Reference		
Retail market	10	3	30.0 (6.7, 65.3)	4.8 (1.1, 21.1)		
Smallholder farm	214	124	57.9 (51.3, 64.6)	10.1 (6.3, 16.3)		
Slaughterhouse	25	5	20.0 (3.1, 36.9)	4.3 (1.3, 13.8)		

Note: aOR, adjusted odds ratio; CI, confidential interval.

## 4 Discussion

Workers in sheep-related occupations often have little or no awareness of the zoonotic transmission risks posed by HEV, even though the virus is a valuable sentinel pathogen for gauging spillover risks at the human-animal-environment interface [31]. ‌While HEV genotype 4, which is frequently detected in sheep, has been extensively reported in farm and market settings and among shepherds and sheep milk cheeseworkers [21], parallel studies on its presence in mutton supply chains and exposed occupational groups remain notably scarce. This shortfall is especially troubling, while sheep are well-established reservoirs of HEV, the risks to workers who handle them remain largely overlooked. Our findings revealed a higher HEV seroprevalence among sheep industry workers (53.0%) compared to the general population (13.3%), indicating occupational exposure as a major risk factor. An aOR of 9.5 (95% CI: 5.9–15.3) indicates a strongly significant association, demonstrating that close and frequent contact with sheep substantially increases the risk of HEV infection. These findings align with international evidence suggesting that zoonotic HEV transmission poses a substantial occupational health threat in both developed and developing regions, particularly for those who are routinely exposed to livestock.

Our analysis has identified several key factors influencing HEV risk among occupationally exposed workers. Notably, prolonged exposure duration, particularly from 5 to 14 years, was associated with markedly increased odds of infection (OR = 10.1, 95% CI: 5.8–17.5), highlighting the cumulative nature of risk in this population. This finding aligns with evidence from multiple research studies. In Europe, studies have demonstrated elevated HEV seroprevalence among swine-exposed occupations: German slaughterhouse workers, meat inspectors, swine farmers, and veterinarians exposed to pigs showed elevated anti-HEV seroprevalance, with slaughterhouse workers having significantly higher rates compared to blood donors [32]. A meta-analysis that synthesized 32 studies confirmed that in Asia, where China was the primary study country, occupationally swine-exposed individuals had a 1.49-fold higher risk of HEV infection (95% CI: 1.35–1.64) than the general population [33]. These collective findings demonstrate that the extended duration of animal contact is a consistent determinant of HEV infection risk across diverse geographic and occupational contexts.

The markedly higher risk among females (OR = 2.7, 95% CI: 1.6–4.8) perhaps reflects gender-specific occupational roles, immunological differences, or distinct health-seeking behaviors. The even greater risk observed in workers on smallholder farms (OR = 9.0, 95% CI: 5.7–14.2) relative to those in retail or slaughterhouse settings may be explained by more direct and prolonged animal contact, less stringent use of personal protective equipment, or poorer hygiene. Although recorded primary occupations showed no direct links to known HEV reservoirs, residual confounding from unmeasured environmental or domestic exposures cannot be ruled out. These findings were consistent with evidence from endemic regions, where small-scale farming is a key driver of zoonotic pathogen transmission.

The high IgG seropositivity (51.8%) coupled with low IgM seropositivity (2.0%) suggests that HEV infections among sheep farmers are predominantly past, rather than acute, indicating widespread prior exposure. This pattern aligns with zoonotic HEV epidemiology, as documented in European and Asian occupational studies. Nevertheless, chronic infection can cause severe sequelae in immunocompromised individuals, and subclinical cases may sustain transmission chains within occupational groups and the wider community.

Globally, HEV is increasingly being recognized as an occupational hazard. This is supported by a recent study in Saudi Arabia, which found that slaughterhouse workers had a significantly higher HEV seroprevalence than a control group of blood donors (49.7% vs. 22.1%, *p* < 0.0001) [34]. Our findings align with this international consensus and extend it to sheep handlers, a group that has received comparatively less attention than swine workers. This is particularly relevant given the expansion of sheep farming worldwide and the potential role of small ruminants as reservoirs for HEV.

Our study has several limitations. First, the cross-sectional study design precludes the causal inferences, although we adjusted for key confounders; residual confounding may persist due to unmeasured variables, such as detailed hygiene practices or non-occupational exposure. Second, as the study did not include viral genotyping or phylogenetic analysis, we could not assess the genetic relatedness of viral strains between sheep and human participants; furthermore, the lack of viral RNA detection limits our ability to confirm active viremia or assess genotype-specific risks. Third, the relatively small sample sizes of slaughterhouse and retail market workers limited the statistical power for robust subgroup analyses and risk evaluations in these occupational populations.

In conclusion, this study provides robust evidence that occupational exposure to sheep significantly increases the risk of HEV infection, with risk influenced by the duration of exposure, gender, and the workplace setting. These findings highlight the urgent need for targeted preventive measures, including zoonotic risk education, improved hygiene protocols and infrastructure, and consideration of vaccination for high-risk populations. Future longitudinal studies are essential to clarify patterns of HEV incidence, assess the long-term health impacts of occupational HEV exposure, and evaluate the effectiveness of interventions to protect this vulnerable workforce.

## Funding information

This work was supported by the Department of Science and Technology of Xinjiang of China (grant no. 2023TSYCTD0017), the 10.13039/501100001809National Natural Science Foundation of China (grant no. 82560656), and the Scientific Research Innovation Team Project for Xinjiang Medical University (grant no. XYD2024C04) and the 14-th Five-Year Plan Distinctive Program of Public Health and Preventive Medicine in Higher Education Institutions of Xinjiang Uygur Autonomous Region.

## CRediT authorship contribution statement

**Wan-Ting Xu:** Writing – original draft, Investigation, Formal analysis. **Yu-Song Ding:** Investigation. **Yang-Gui Chen:** Investigation. **Ya-Hui Feng:** Investigation, Formal analysis. **Jun Li:** Writing – review & editing, Supervision. **Ji-Guo Jin:** Investigation. **Xiang-Nan Wei:** Investigation. **Fan Wu:** Investigation. **Xing-Yu Wang:** Investigation. **Xing-Tao Dang:** Investigation. **Guo-Wu Zhang:** Investigation. **Xue-Ying Xiang:** Investigation. **Fu-Ye Li:** Investigation, Funding acquisition. **Wen-Bao Zhang:** Writing – review & editing, Investigation, Funding acquisition. **Jian-Yong Wu:** Writing – review & editing, Writing – original draft, Supervision, Resources, Project administration, Methodology, Investigation, Funding acquisition, Data curation, Conceptualization.

## Declaration of competing interest

The authors declare no conflict of interest.

## Data Availability

Data will be made available on request.
